# Protein kinase FAM20C—when subcellular localization matters

**DOI:** 10.1002/1873-3468.70172

**Published:** 2025-09-19

**Authors:** Francesca Noventa, Mauro Salvi

**Affiliations:** ^1^ Department of Biomedical Sciences University of Padova Italy

**Keywords:** acidophilic kinase, kinase specificity, protein phosphorylation

## Abstract

Protein kinases achieve signaling specificity through consensus sequence recognition and subcellular localization. Although motif analysis can predict potential substrates, this method is insufficient if the spatial context in which the kinase operates is not considered. FAM20C, an acidophilic kinase that is primarily localized to the Golgi lumen, is an example of this. Its preference for the SXE motif informs substrate selection, but only within compartments accessible to the enzyme. Assigning substrates based solely on motif presence can be misleading and requires integrative experimental strategies to determine whether specific conditions enable kinase–substrate proximity.

## Graphical abstract

FAM20C is a Golgi‐resident kinase that phosphorylates proteins along the entire secretory pathway. The presence of potential FAM20C substrates in the cytoplasm or nucleus raises the question of how the kinase and its substrates encounter each other.

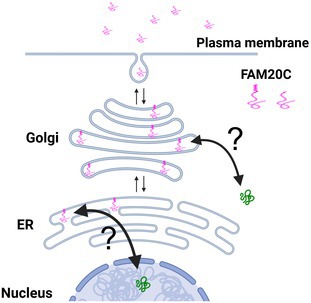

## Abbreviations

**AA**, amino acids

**ER**, endoplasmic reticulum

**GCK**, Golgi casein kinase

**HIBD**, hypoxic–ischemic brain damage

**Fig. 1:**
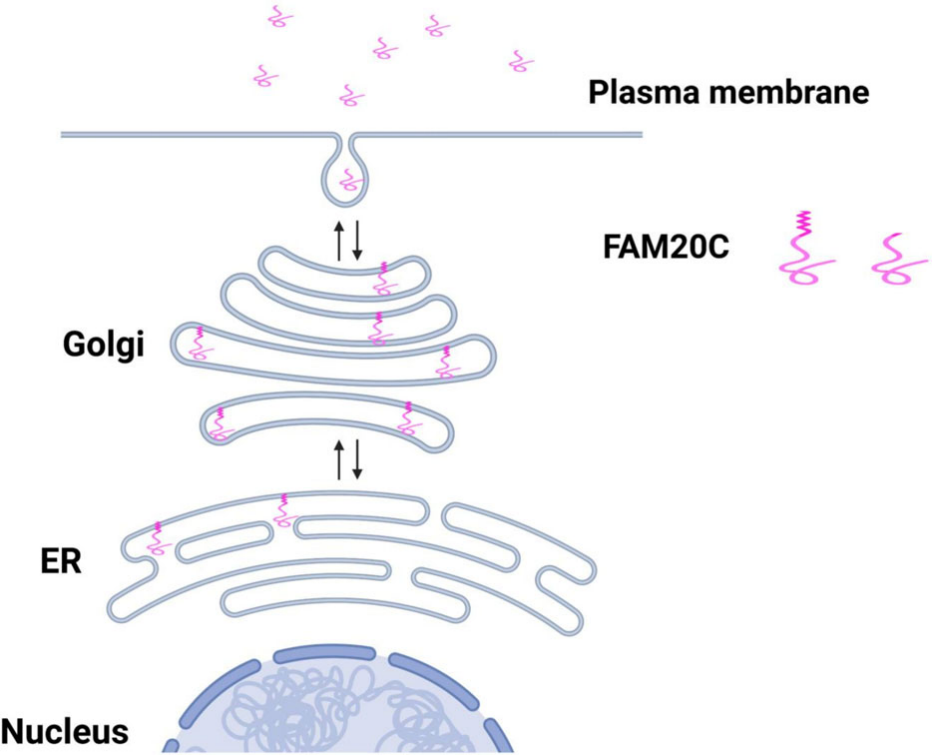
Fam20C subcellular localization. The picture shows FAM20C subcellular localization with or without the N‐terminal transmembrane domain. The picture has been produced using BioRender.

The human genome encodes more than 500 protein kinases, each characterized by distinct consensus sequence preferences and subcellular localization patterns, features that ensure the spatial and temporal fidelity of signal transduction into specific biological outcomes. Among these, protein kinase FAM20C stands out as a notable example of how localization and substrate specificity converge to define kinase function. Before its molecular identification in 2012 [1, 2], FAM20C, formerly known as Golgi casein kinase (GCK), due to its apparent localization to the Golgi compartments, was classified as an acidophilic kinase, with a distinctive consensus sequence of SXE [3]. The kinase indeed shows a strong preference for phosphorylating Ser over Thr, and the glutamic acid at the +2 position can also be substituted by a phosphorylated serine, though with lower efficiency [3]. This motif differs from the canonical (S/T)XX(E/D) sequence recognized by the well‐characterized protein kinase CK2 [3], whereas a phosphoresidue in the +3 position can partly substitute Glu or Asp [4]. In 2010, an analysis of phosphosites present in extracellular proteins revealed that the majority contained the SXE motif [5], suggesting that GCK might play a key role in modifying proteins destined for the extracellular space, a compartment topologically equivalent to the Golgi lumen. The identification of GCK as FAM20C [1, 2] enabled experimental validation of its broader function in extracellular protein phosphorylation [6]. FAM20C was suggested to be a type II transmembrane protein localized in the Golgi, composed of a very short cytoplasmic N terminus (1–10 AA), a single‐pass transmembrane domain (11–30 AA), and a C‐terminal kinase domain located within the Golgi lumen. Proteolytic cleavage of the FAM20C between residues 92 and 93 by the Golgi‐resident protease S1P promotes its secretion [7]. Further studies revealed that FAM20C co‐immunoprecipitates with several proteins residing in the endoplasmic reticulum (ER), suggesting that many of its substrates may localize within the ER [8, 9], a compartment that, while earlier in the secretory pathway, remains topologically continuous with the Golgi lumen. This raised the possibility that FAM20C phosphorylates substrates either within the ER prior to Golgi trafficking, through retrograde transport from the Golgi to the ER, or via dynamic shuttling of substrates between the two compartments, as suggested for proteins, such as Ero1α [8]. Together, these findings support the idea that FAM20C's functional role extends beyond the Golgi, potentially phosphorylating proteins throughout the entire secretory pathway (Fig. 1).

Feng et al. in their paper recently published in Cell Proliferation [10] explore the role of FAM20C in hypoxic–ischemic brain damage (HIBD) and its implications for neurodevelopmental disorders. The research is significant because HIBD, resulting from perinatal asphyxia or hypoxia, remains a leading cause of neonatal mortality and long‐term neurological disabilities.

The study shows that FAM20C expression is reduced in ischemic hippocampal neurons post‐HIBD (mRNA and protein), with the most pronounced decrease at 24 h [10]. Loss of FAM20C impairs neuronal differentiation and promotes proliferation, indicating a possible switch from differentiation to survival programs under hypoxic stress. Behavioral experiments further support a protective role for FAM20C, as its overexpression partially rescues cognitive deficits [10].

By combining co‐immunoprecipitation experiments with analysis of the FAM20C consensus sequence at phosphoserine sites in the co‐immunoprecipitated proteins, the authors identified six potential FAM20C substrates, including KAP1, YTHDC1, ADAR, and SAFB, all cytoplasmic/nuclear proteins with the only exception of DAGLA, which localizes in the cell membrane. KAP1 phosphorylation by FAM20C at S473 and S489 was confirmed using Phos‐tag assays. Co‐localization of FAM20C with KAP1 and YTHDC1 was also demonstrated via immunofluorescence [10]. The functional relevance of this interaction is underscored by KAP1's known roles in transcriptional repression, neurogenesis, and cognitive function. The study proposes that FAM20C, by phosphorylating KAP1, negatively affects the formation of the YTHDC1–NCL–KAP1–LINE1 RNA complex, impacting H3K9me3‐mediated epigenetic silencing. The upstream regulatory pathway involves E2F4, a transcription factor downregulated under hypoxic conditions, whose reduced expression leads to decreased FAM20C levels [10].

These findings might offer new insights into the role of FAM20C in the developing brain and raise the possibility of therapeutic strategies aimed at restoring FAM20C activity in HIBD.

However, an important aspect that must be carefully considered is FAM20C's well‐established subcellular localization. FAM20C is not a cytoplasmic kinase. FAM20C is predominantly localized to the Golgi apparatus, with the kinase domain in the lumen, is secreted extracellularly, and its known substrates reside exclusively within the secretory pathway. In contrast, most of the interactors/potential substrates identified in this study, including the transcriptional co‐repressor protein KAP1, are cytoplasmic or nuclear proteins.

This discrepancy raises a fundamental question. How could FAM20C directly interact with and phosphorylate proteins, such as KAP1 and YTHDC1, which are localized in the nucleus or cytoplasm? Although the presence of phosphoresidues containing the SXE motif in these proteins supports potential phosphorylation by FAM20C, motif‐based predictions alone are insufficient to establish bona fide kinase–substrate relationships, particularly when the predicted substrates reside in cellular compartments where the kinase is not known to function.

This inconsistency indicates that additional investigation is required to substantiate the claim that FAM20C directly phosphorylates these proteins. Several plausible but unaddressed scenarios could reconcile this apparent contradiction. One possibility is that FAM20C undergoes hypoxia‐induced mislocalization to the cytosol or nucleus, although there is currently no direct evidence supporting this. Alternatively, substrates such as KAP1 may transiently access the secretory pathway, but this has yet to be demonstrated. Another unexplored possibility is alternative translation initiation, which would generate an isoform lacking the transmembrane region and localizing outside the secretory pathway. Finally, and perhaps the most plausible scenario is an indirect mechanism where FAM20C phosphorylates an intermediary protein within the secretory pathway, which in turn modulates nuclear factors. All these possibilities remain unexplored in the present study and were not considered, as the subcellular localization of FAM20C was not taken into account.

More broadly, this case highlights an important general principle. While kinase consensus motifs provide useful clues, they must be interpreted within the spatial context of kinase activity.

In summary, while this study provides exciting new avenues for understanding FAM20C's role in neurodevelopment and hypoxic injury, future work should aim to resolve the kinase/substrates localization conundrum.

## Conflict of interest

The authors declare no conflict of interest.

## Author contributions

F.N.: Writing – original draft; writing – review and editing. M.S.: Conceptualization; writing – original draft; writing – review and editing.
